# 中国复发难治性急性髓系白血病诊疗指南（2023年版）

**DOI:** 10.3760/cma.j.issn.0253-2727.2023.09.002

**Published:** 2023-09

**Authors:** 中华医学会血液学分会白血病淋巴瘤学组

修订要点：

• 在复发难治性急性髓系白血病（AML）的治疗原则中不再以年龄作为首要分层因素，而是以能否耐受强化疗作为首要分层因素。

• 在强化疗方案中新增靶向药物联合强化疗相关方案。

• 新增复发难治性AML诊疗流程图。

一、复发、难治性AML诊断标准

1. 复发性AML诊断标准：AML完全缓解（CR）后外周血再次出现白血病细胞或骨髓中原始细胞≥5％（除外巩固化疗后骨髓再生等其他原因）或髓外出现白血病细胞浸润。

2. 难治性AML诊断标准：经过标准方案治疗两个疗程无效的初治病例；CR后经过巩固强化治疗，12个月内复发者；在12个月后复发且经过常规化疗无效者；两次或两次以上复发者；髓外白血病持续存在者。

二、复发、难治性AML预后评估

AML复发后生存率低的相关因素包括持续缓解时间较短（6～12个月）、诊断时检出预后不良核型或不良基因、年龄较大（>45岁）、既往造血干细胞移植治疗史[1]。此外，体能状态差、共病存在等也与预后不良相关。

对于复发难治性AML患者应该再次进行细胞遗传学和分子遗传学异常的评估（如染色体分析、靶向外显子测序、转录组测序等），以明确是否存在或新出现某些特殊染色体异常、基因突变或融合基因，为再次治疗方案选择提供帮助。

三、复发、难治性AML治疗原则

复发难治性AML异质性强，预后极差，5年生存率约10％。

符合条件的复发难治性AML患者应首选参加临床试验，其他治疗选择主要包括靶向治疗或化疗后序贯异基因造血干细胞移植（allo-HSCT）。靶向治疗是近年来AML领域的热点，不断上市的靶向药物为一些复发难治性AML患者带来再次缓解的机会，对于此类患者应积极寻找可靶向的突变基因。对于原发耐药或者早期复发且无法缓解的患者，也可直接采取allo-HSCT作为挽救治疗措施。免疫治疗（主要包括CAR-T细胞治疗和双靶点抗体治疗）获益/风险评估尚不充分，仍处于试验阶段。

在选择试验及治疗方案时，应充分考虑患者遗传学异常特点、复发时间、个体因素（如年龄、体能状态、合并症、既往治疗方案）等特点以及患方的治疗意愿。复发难治性AML的诊疗流程见图1。

**图1:**
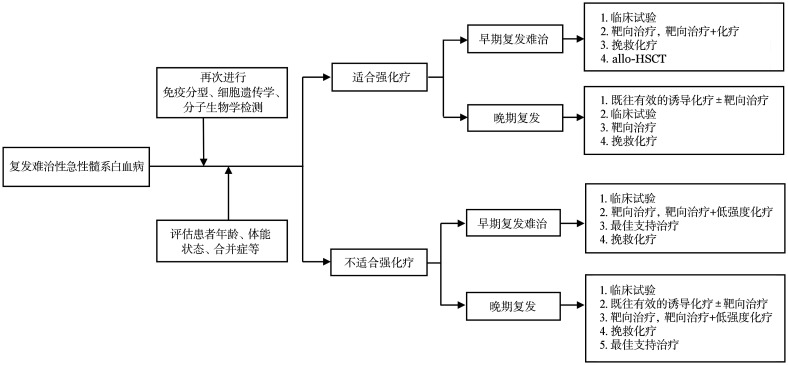
复发难治性急性髓系白血病诊疗流程图

1. 适合强化疗的患者：患者一般状态好，耐受性佳。

（1）早期复发者（缓解后12个月以内复发者）：①临床试验；②靶向药物治疗或者靶向药物联合化疗；③挽救化疗；④直接进行allo-HSCT。

（2）晚期复发者（缓解后12个月以上复发者）：①重复初始有效的诱导化疗方案或在原有诱导化疗方案基础上联合靶向药物，再次缓解后建议行allo-HSCT；②临床试验；③靶向药物治疗；④挽救化疗。

（3）难治性患者按照早期复发者方案处理。

2. 不适合强化疗的患者：患者年龄≥75岁或因体能状态、合并症等因素不适合强诱导化疗患者。

（1）早期复发者：①临床试验；②靶向药物治疗或者靶向药物联合低强度治疗；③最佳支持治疗；④挽救化疗，缓解后如体能状态好转可以考虑allo-HSCT。

（2）晚期复发者：①临床试验；②重复初始有效的诱导方案或在原有诱导方案基础上联合靶向药物；③靶向药物治疗或者靶向药物联合低强度治疗；④最佳支持治疗（用于不能耐受或者不愿意进一步治疗的患者）。

（3）难治性患者按照早期复发者方案处理。

四、复发、难治性AML的治疗方案

在前述治疗原则的基础上，本指南延续上一版治疗指南，突出新的靶向治疗药物的重要地位，并基于新的循证医学证据，结合患者耐受情况，推荐针对复发难治性AML患者的治疗方案。需要注意的是，以下方案涉及药物均为国内已上市药物，对于国内未上市药物的相关方案暂未列入其中。

1. 靶向药物±去甲基化药物：如患者检测有FLT3突变或者IDH1突变，可使用针对特异性靶点的靶向药物（如吉瑞替尼、索拉非尼、艾伏尼布等）。维奈克拉为BCL-2抑制剂，可广泛用于AML患者。

（1）靶向FLT3突变阳性：

①FLT3-ITD：吉瑞替尼（Gilteritinib）[2]–[3]：Ⅰ型口服FLT3抑制剂，治疗剂量为120 mg/d；联合阿扎胞苷75 mg/m^2^，第1～7天；或地西他滨20 mg/m^2^，第1～5天（证据等级1a）。

索拉菲尼（Sorafenib）：Ⅱ型口服FLT3抑制剂，治疗效量为400 mg，每日2次；联合阿扎胞苷75 mg/m^2^，第1～7天；或地西他滨20 mg/m^2^，第1～5天（证据等级2a）。

②FLT3-TKD：吉瑞替尼：治疗剂量120 mg/d（证据等级1a）。

（2）靶向IDH1突变阳性：

艾伏尼布（Ivosidenib）[4]：治疗剂量为500 mg/d；可联合去甲基化药物，去甲基化药物剂量及用法同上（证据等级2a）（仅针对IDH1突变的患者）。

（3）BCL-2抑制剂：

①维奈克拉（Venetoclax）+去甲基化药物：维奈克拉第1天100 mg，第2天200 mg，第3天开始400 mg，直至第28天；合并使用唑类药物时需调整剂量；去甲基化药物剂量及用法同上。

②维奈克拉+其他靶向药物±去甲基化药物：目前不同的两药/三药联合的靶向治疗方案在不断的探索研究中，并且取得了良好的疗效，如维奈克拉+吉瑞替尼[5]（对于FLT3突变患者）、维奈克拉+阿扎胞苷+吉瑞替尼[6]（对于FLT3突变患者）、维奈克拉+艾伏尼布±阿扎胞苷[7]（对于IDH1突变患者）等。

2. 强化疗方案：既往强化疗方案主要包含以嘌呤类似物（如氟达拉滨、克拉屈滨）为主的方案，缓解率为30％～45％，中位生存期8～9个月。近两年在化疗基础上联用靶向药物在复发难治性AML患者中显示了较高的缓解率，可达40％～70％，成为临床实践的重要选择。

（1）化疗药物联合靶向药物：

①维奈克拉+FLAG-IDA方案[8]：维奈克拉400 mg/d，14 d；氟达拉滨（Fludarabine）30 mg/m^2^，第2～6天；阿糖胞苷（Ara-C）1.5～2.0 g/m^2^，第2～6天；去甲氧柔红霉素（Idarubicin, IDA）6 mg/m^2^，第4～5天；G-CSF 5 µg/kg，第1～7天。

②维奈克拉+阿扎胞苷+高三尖杉酯碱（Homoharringtonine, HHT）[9]：维奈克拉100 mg第1天，200 mg第2天，400 mg第3～14天；阿扎胞苷75 mg/m^2^，第1～7天；HHT 1 mg/m^2^，第1～7天。

此外，如维奈克拉+克拉屈滨+低剂量Ara-C（LDAC）[10]、维奈克拉+阿扎胞苷+西达本胺[11]、维奈克拉+阿扎胞苷+LDAC[12]等多种联合方案也在小队列复发难治性AML患者的临床探索中显示良好的疗效，等待后续更大队列的疗效验证。而维奈克拉+DA[13]、维奈克拉+CLIA[14]等维奈克拉与标准化疗的联合方案在初治AML患者中显示良好的疗效，在复发难治性AML中的疗效等待后续的验证。

（2）传统化疗方案：

①HAA（HAD）：HHT 2 mg/m^2^，第1～7天（或HHT 4 mg/m^2^，分2次给药，第1～3天）；Ara-C 100～200 mg/m^2^，第1～7天；阿柔比星（Aclarubicin，Acla）20 mg/d，第1～7天［或柔红霉素（Daunorubicin, DNR）45 mg/m^2^第1～3天］。

②中大剂量Ara-C±蒽环类药物：Ara-C 1～3 g/m^2^，每12 h 1次，第1、3、5天；联合DNR 45 mg/m^2^或IDA 10 mg/m^2^，第2、4、6天。或Ara-C（既往没有用过大剂量Ara-C的患者可以选择）3 g/m^2^，每12 h 1次，第1～3天。

③CLAG±IDA/米托蒽醌（Mitox）方案：克拉屈滨5 mg/m^2^，第1～5天；Ara-C 1～2 g/m^2^，克拉屈滨用后4 h使用，第1～5天，静脉滴注3 h；G-CSF 300 µg/m^2^，第0～5天（WBC>20×10^9^/L暂停）；IDA 10～12 mg/m^2^，第1～3天，或Mitox 10～12 mg/m^2^，第1～3天。

④FLAG±IDA：氟达拉滨30 mg/m^2^，第1～5天；Ara-C 1～2 g/m^2^，氟达拉滨用后4 h使用，第1～5天，静脉滴注3 h；G-CSF 300 µg/m^2^，第0～5天；IDA 10～12 mg/m^2^，第1～3天。

⑤EA±Mitox：依托泊苷100 mg/m^2^，第1～5天；Ara-C 100～150 mg/m^2^，第1～7天；Mitox 10 mg/m^2^，第1～5天。

⑥CAG：G-CSF 300 µg/m^2^，每12 h 1次，第0～14天；Acla 20 mg/d，第1～4天；Ara-C 10 mg/m^2^，皮下注射，每12 h 1次，第1～14天。

3. 非强化疗方案：

①维奈克拉+去甲基化药物：维奈克拉第1天100 mg，第2天200 mg，第3天开始每天400 mg直至第28天；阿扎胞苷75 mg/m^2^，皮下注射，第1～7天或者地西他滨20 mg/m^2^，皮下注射，第1～5天，直至出现疾病进展或者患者不可耐受（证据等级2a）。

② 维奈克拉+LDAC：维奈克拉第1天100 mg，第2天200 mg，第3天400 mg，第4天开始每天600 mg直至第28天；Ara-C 10 mg/m^2^，皮下注射，每12 h 1次，第1～10天，直至出现疾病进展或者患者不可耐受（证据等级2a）。

③去甲基化药物（HMA）：阿扎胞苷75 mg/m^2^，第1～7天，28 d为1个疗程，直至患者出现疾病恶化或严重不良反应；或地西他滨20 mg/m^2^，第1～5天，28 d为1个疗程，直至患者出现疾病恶化或严重不良反应（证据等级2a）。

④LDAC：Ara-C 10 mg/m^2^，皮下注射，每12 h 1次，第1～14天（证据等级2b）。

4. allo-HSCT：复发难治性AML患者获得缓解后如条件许可应尽早进行allo-HSCT。对于某些患者，尤其是原发耐药或早期复发且无法缓解的患者，也可以直接采取allo-HSCT作为挽救治疗措施（证据等级2b）。

5. 免疫治疗：主要包括CAR-T细胞治疗和双靶点抗体治疗。目前AML的CAR-T细胞治疗已有多种作用靶点报道，如CD33、CD123、CLL-1、CD70等，但尚无大规模数据结果报道，且相关产品均在试验中。
